# *In Vivo* 7T MRI of the Non-Human Primate Brainstem

**DOI:** 10.1371/journal.pone.0127049

**Published:** 2015-05-12

**Authors:** Laura M. Zitella, YiZi Xiao, Benjamin A. Teplitzky, Daniel J. Kastl, Yuval Duchin, Kenneth B. Baker, Jerrold L. Vitek, Gregor Adriany, Essa Yacoub, Noam Harel, Matthew D. Johnson

**Affiliations:** 1 Department of Biomedical Engineering, University of Minnesota, Minneapolis, Minnesota, United States of America; 2 Center for Magnetic Resonance Research, University of Minnesota, Minneapolis, Minnesota, United States of America; 3 Department of Neurology, University of Minnesota, Minneapolis, Minnesota, United States of America; 4 Institute for Translational Neuroscience, University of Minnesota, Minneapolis, Minnesota, United States of America; University Health Network and University of Toronto, CANADA

## Abstract

Structural brain imaging provides a critical framework for performing stereotactic and intraoperative MRI-guided surgical procedures, with procedural efficacy often dependent upon visualization of the target with which to operate. Here, we describe tools for *in vivo*, subject-specific visualization and demarcation of regions within the brainstem. High-field 7T susceptibility-weighted imaging and diffusion-weighted imaging of the brain were collected using a customized head coil from eight rhesus macaques. Fiber tracts including the superior cerebellar peduncle, medial lemniscus, and lateral lemniscus were identified using high-resolution probabilistic diffusion tractography, which resulted in three-dimensional fiber tract reconstructions that were comparable to those extracted from sequential application of a two-dimensional nonlinear brain atlas warping algorithm. In the susceptibility-weighted imaging, white matter tracts within the brainstem were also identified as hypointense regions, and the degree of hypointensity was age-dependent. This combination of imaging modalities also enabled identifying the location and extent of several brainstem nuclei, including the periaqueductal gray, pedunculopontine nucleus, and inferior colliculus. These clinically-relevant high-field imaging approaches have potential to enable more accurate and comprehensive subject-specific visualization of the brainstem and to ultimately improve patient-specific neurosurgical targeting procedures, including deep brain stimulation lead implantation.

## Introduction

Structural brain imaging has become an important tool for guiding neurosurgical procedures, including microelectrode mapping, catheter insertion, ablation, and deep brain stimulation (DBS) lead implantation [1, 2]. Image-based targeting approaches can be especially useful when the dimensions and locations of the neuroanatomical targets vary amongst patients [3] and when the targets are small and embedded within complex networks of nuclei and fiber tracts, which may influence clinical outcomes when affected by the neurosurgical treatment [4]. Recent investigational applications of electrical stimulation within the brainstem to treat parkinsonian freezing of gait [5, 6], relieve central pain [7], and restore hearing [8] underscores the need for more refined image-based targeting techniques of DBS lead implants in the context of the brainstem. This is especially relevant given that millimeter-scale implantation inaccuracies often result in lower stimulation thresholds for evoking side effects than for delivering therapy [9–12].

In practice, however, imaging detailed neuroanatomy of the brainstem with conventional MR scanners (1.5-3T) has been difficult [13] due to overall lack of contrast, small region of interest, and ambiguous borders between nuclei and fiber tracts [4, 14]. Higher field strength scanners have yielded higher-resolution images at 7T [15, 16] and 8T [17], while alternative sequences have provided higher contrast images in parts of the brainstem [18–26]. Diffusion-weighted imaging (DWI) at 3T [27–29] and 7T [30–32] and diffusion tractography at 1.5T [33–35] and 3T [14, 36] have been useful to identify fiber tracts within the brainstem non-invasively; however, these techniques do not include probabilistic tractography and have not been previously validated. Other studies utilizing very high resolution *ex vivo* imaging and histology have been able to identify regions within the human brainstem [37] and validate tractography [38]. However, *ex vivo* imaging is not practical for direct targeting applications so there is need to further integrate *in vivo* high field imaging and high-resolution probabilistic tractography approaches for visualization of structures within the brainstem.

While the development of *in vivo* high field imaging [39, 40] with advanced diffusion weighted imaging sequences [32, 41, 42] has potential to increase the spatial resolution of imaging the brainstem [43], there is also a necessity to validate the contrast maps [44] and quantify how they vary amongst subjects [35]. For example, current *in vivo* MRI-based techniques to localize the pedunculopontine nucleus (PPN) in the human brainstem have utilized an atlas to predict the coordinates of the PPN in relation to the 4^th^ ventricle and the contrast of proton-density MRI to estimate the general area of the PPN. The atlas-based methods have produced reasonable localization in the lateral and anteroposterior coordinates (within 0.5 mm) but large inaccuracies in the rostrocaudal coordinates (3.3 mm) [4].

Here, we show that a multi-modal imaging approach using 7T MRI *in vivo* enables accurate identification of the PPN, inferior colliculus (IC), and periaqueductal gray (PAG) as confirmed with histology in two subjects. The acquired dataset enabled: 1) investigating what contrast exists in the non-human primate brainstem using high-field 7T susceptibility-weighted imaging, 2) developing methods to identify structures not directly visible even with high-field MRI, 3) generating probabilistic fiber tractography of the brainstem, 4) assessing the anatomical variability of brainstem structures across eight rhesus macaques, and 5) comparing the nuclei and fiber tract reconstructions to post-mortem histology. Improvements in the visualization of anatomical targets using these tools hold promise for more accurate subject-specific surgical targeting of interventions in the brainstem [9] ultimately influencing the clinical outcomes of neurosurgical interventions in this region of the brain.

## Materials and Methods

### Data Acquisition

Eight rhesus macaque monkeys (*macaca mulatta*, *7 females*, *1 male*, Table 1) were scanned at the Center for Magnetic Resonance at the University of Minnesota, using a passively shielded 7T magnet (Magnex Scientific) operating with a Siemens console and head gradient insert capable of 80 mT/m and a slew rate of 333 mT/m/s. A radio frequency head coil, consisting of 16 transmit and 16+6 receive channels, with 4 smaller element coils positioned on top of the head for higher sensitivity and 2 ear-loop coils to enhance signal detection from brainstem structures, was designed specifically for primate studies [45]. All procedures were approved by the Institutional Animal Care and Use Committee of the University of Minnesota and complied with United States Public Health Service policy on the humane care and use of laboratory animals. Animals were housed individually in a Primate Products Enhanced Environment Housing System (12/12 hour light dark cycle) in the Research Animal Resources facility of the University of Minnesota. The animals were given a range of environmental enrichment (e.g. toys, mirrors, TV), provided with water *ad libitum*, and given a range of food options including fresh fruit and vegetables. All efforts were made to provide good care and alleviate unnecessary discomfort, and no adverse events occurred. Animals were anesthetized with isoflurane (2.5%) during the imaging sessions and monitored for depth of anesthesia. At the conclusion of the study and in order to validate the imaging data, two animals were randomly chosen to be deeply anesthetized with sodium pentobarbital and perfused with a fixative solution containing 4% paraformaldehyde, consistent with the recommendations of the Panel on Euthanasia of the American Veterinary Medical Association.

**Table 1:** Subject characteristics and imaging protocols (*iso*: *isometric*).

Subject	Gender	Age	Resolution (mm)		
			T1-W	T2-W	SWI
**M1**	F	22	0.667 iso	0.4x0.4x0.7	0.4 iso
**M2**	F	22	0.667x0.667x0.33	0.4x0.4x0.8	0.4 iso
**M3†** *	F	18	0.5 iso	0.33 iso	0.33 iso
**M4** *	F	14	0.5 iso	0.33 iso	0.33 iso
**M5†**	F	13	0.5 iso	0.5 iso	0.4 iso
**M6†**	F	10	0.667x0.667x0.7	0.4x0.4x0.8	0.4 iso
**M7**	F	9	0.5 iso	0.5 iso	0.4 iso
**M8†**	M	4	0.5x0.5x0.249	0.4x0.4x0.8	0.33 iso

† tractography performed

* histological confirmation

Imaging sequences included T1-weighted imaging (T1-W), T2-weighted imaging (T2-W), susceptibility-weighted imaging (SWI), and diffusion-weighted imaging (DWI). T1-W images and T2-W images were acquired with a 3D-MPRAGE sequence and a 2D turbo spin echo sequence, respectively, with the resolutions shown in Table 1. SWI was acquired with a 3D flow-compensated gradient echo sequence using a FOV of 128 x 96 x 48 mm^3^, matrix size of 384 x 288 x 144 (0.33–0.4 mm isotropic resolution), TR/TE of 35/29 ms, flip angle of 15°, BW of 120 Hz/pixel, and acceleration factor of 2 (GRAPPA) along the phase-encoding direction. DWI was acquired with a single refocused 2D single-shot spin echo EPI sequence [46] using a FOV of 128 x 84 x 99 mm^3^, matrix size of 128 x 84 x 50 (1 mm isotropic resolution), TR/TE of 3500/53 ms, BW of 1860 Hz/pixel, and an acceleration factor of 3 (GRAPPA). Diffusion-weighted images (b-value = 1500 s/mm^2^) were acquired with diffusion gradients applied along 142 uniformly distributed directions. Fifteen additional non-diffusion-weighted images (b = 0 s/mm^2^) were also acquired. To correct for geometric distortions in the EPI images due to magnetic field inhomogeneity we utilized TOPUP [47] in FSL. This technique exploits multiple non-diffusion-weighted (b0) scans with opposite (anterior-posterior and posterior-anterior) phase-encoding directions to calculate and compensate for the deformation field.

### Nonlinear Atlas Registration

**Fig 1:**
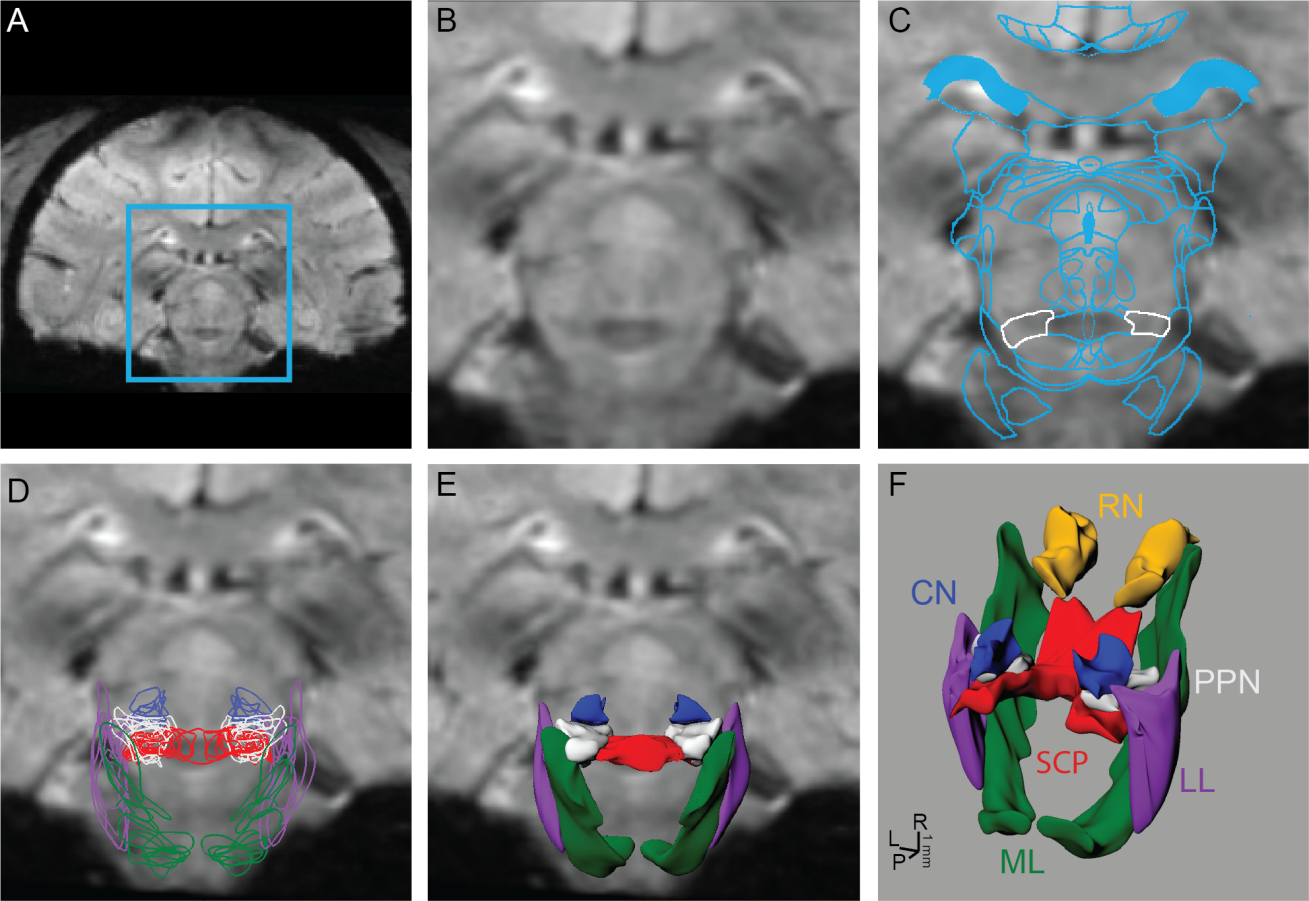
Process for reconstructing brainstem nuclei and fiber tracts in 3D from 7T MRI. The brainstem region outlined in blue (A) was cropped (B) from each coronal 7T SWI MR image. (C) An affine deformation algorithm based on user-defined seed points was used to warp contours from a rhesus macaque brain atlas to the MRI of each subject. The PPN is outlined in white. (D) Algorithm-defined contours from nuclei and fiber tracts within brainstem were outlined on each slice and then (E, F) lofted to create surface renderings.

To identify nuclei and fiber tracts that were not visible on the MRI, a rhesus macaque brain atlas [48] was registered and nonlinearly warped to each subject’s MRI volume, which was aligned in AC-PC space (Analyze) and resliced in the coronal plane. The algorithm (MATLAB) used a nonlinear affine transformation [49, 50] to individually warp 2D atlas slices to corresponding MRI slices. The first and last atlas plates of the desired warped region were matched identically to coronal MRI slices, and the remaining slices were generated from the existing MRI to match the atlas plates exactly. The slices were cropped to include only the brainstem to reduce computational time of the warping algorithm (Fig 1A and 1B), which solved for the transformation that minimized the distance between manually-defined seed points on an atlas image with those placed on an MR image (Fig 1C). A fold-back control feature was added in cases when the Jacobian of the transformation function was negative, in which case the warping procedure was compartmentalized into a series of smaller partial deformations to avoid the sign change. The resultant deformed atlas images were imported sequentially into a non-uniform rational B-spline modeling program (Rhinoceros) to generate 3D surface reconstructions of the individual nuclei and fiber tracts [51, 52] (Fig 1D–1F).

### Probabilistic Diffusion Tractography

In order to obtain accurate 3D visualization of the fiber pathways within the brainstem, we leveraged the warped slices described above to guide the seed points for probabilistic diffusion tractography in FSL in four subjects (M3, M5, M6, and M8) [53–55]. SWI DICOM image sets were converted to NIfTI files (dcm2nii DICOM to NIFTI converter) and imported into FSL. The cranium was removed from the images using the brain extraction tool (BET) in FSL [56]. For all subjects, *flirt* linear registration tool in FSL with 7 degrees of freedom (DOF) was able to obtain a sufficient alignment between the SWI data and diffusion data [57–59], with the latter undergoing a pre-processing routine using the *bedpostx* command to estimate diffusion parameters. An inter-modal cost function (correlation ratio and mutual information-based options) was used because the two images were of different modalities. The output transformation matrix was used to transform the coordinates of objects between SWI and DWI spaces.

**Fig 2:**
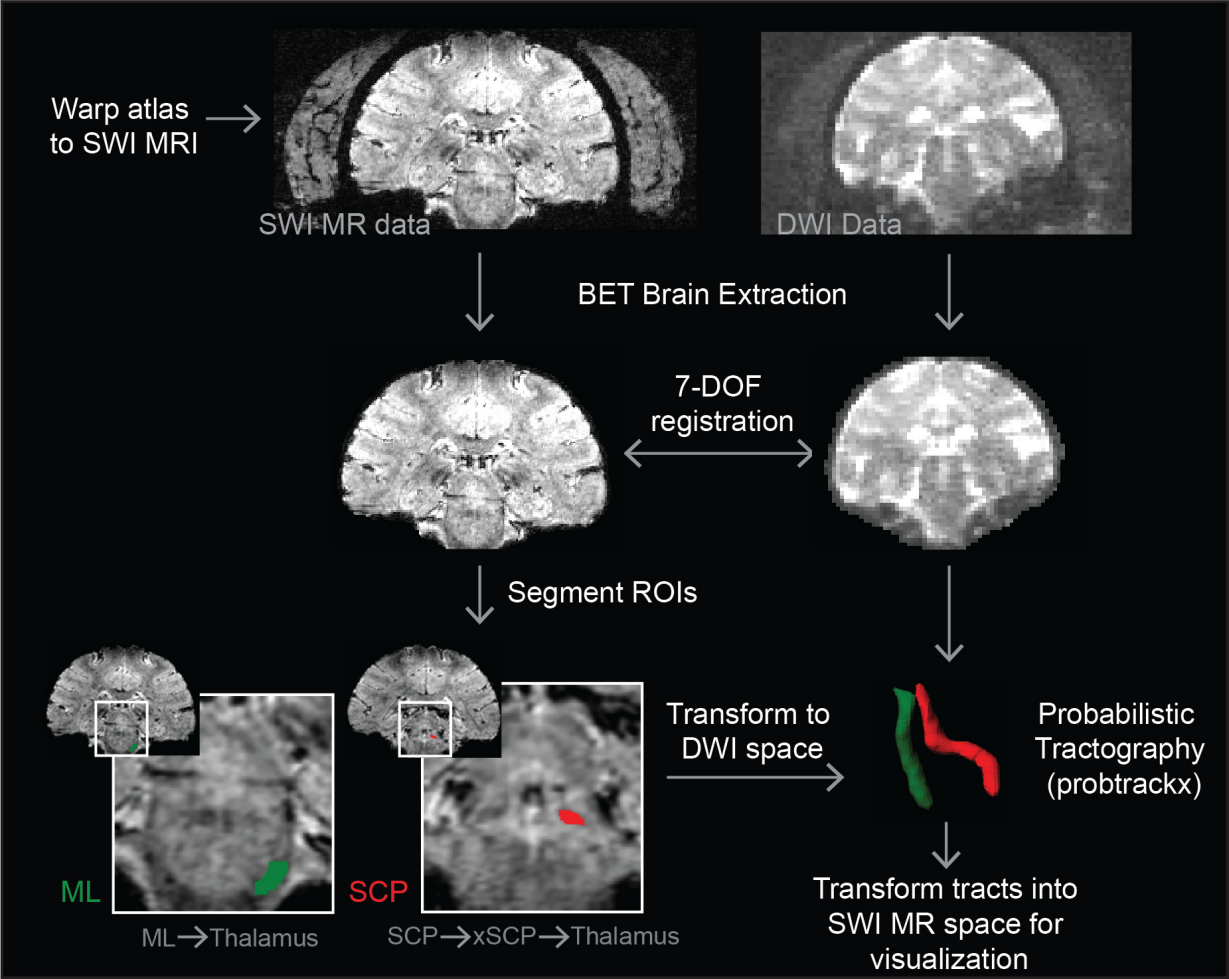
Process to compute subject-specific diffusion tractography. The method combines the warping algorithm and diffusion tractography methods in FSL to identify fiber tracts. Colored regions are the segmented ROIs used in FSL to identify both ML and SCP tracts.

Masks were created in the SWI data in FSLView by manually highlighting pixels where a particular tract began in the caudal brainstem or cerebellum. For the superior cerebellar peduncle (SCP) and medial lemniscus (ML) tracts, a mask of the entire thalamus was segmented manually in FSL and used as a waypoint for the tractography analysis. This ensured that the tracts were not overly guided and that the tractography results could be evaluated for how selectively they projected to their functionally-specific region of thalamus. This process is shown for the placement of the seed mask in the caudal pons to run the tractography algorithm for the ML and its projection into the ventral posterolateral pars caudalis nucleus of thalamus (ventralis caudalis in humans) (Fig 2). In order to reconstruct the portion of the lateral lemniscus (LL), a seed mask was placed just dorsal to the medial lemniscus seed mask with a waypoint mask segmented in the medial geniculate body (MGB) by way of the inferior colliculus (IC). Tractography of the SCP was more complicated due to its decussation in the midbrain. From the decussation, the majority of the crossed fibers are known to ascend to the red nucleus and either terminate there or continue rostrally to the motor nucleus of thalamus [60]. In order to identify this pathway, the seed masks were placed in the posterior pons with way point masks placed at the decussation of the SCP and the entire contralateral thalamus. In two animals (M6 and M8), an additional waypoint in the red nucleus (RN) was used to better identify the SCP tract.

The masks were then transformed into DWI space using the inverse of the transformation matrix calculated using *flirt*. To compute the tractography, each mask was specified as a seed point mask or a waypoint mask in the *probtrackx* command. The resultant NIfTII file, the output of *probtrackx*, was then transformed back into SWI space for visualization purposes. A threshold was applied to the tracts (Amira, Hillsboro, OR), and AC-PC alignment was used to align the warped nuclei with the tracts as a validation of both the tracts and the warping algorithm.

### Immunohistochemistry

Following completion of all imaging studies, monkey M3 and M4 were deeply anesthetized and given a lethal dose of sodium pentobarbital (100 mg/kg, i.v.). Transcardial perfusion consisted of 0.9% NaCl at room temperature (r.t.) delivered at a rate of approximately 50 ml/min for 40 min followed by 4% paraformaldehyde at 4°C delivered at the same rate for 60 min. The brain was removed and post-fixed with 4% paraformaldehyde in 25mM phosphate buffered saline, pH 7.4 (PBS) at 4°C for 7 days. After fixation, the brain was blocked and cryoprotected in 15% sucrose in PBS at 4°C. Coronal sections, 50 μm thick were cut using a freezing microtome and stained using immunohistochemistry.

The immunohistochemical method used for M3 and M4 was carried out on free-floating sections using avidin-biotin-peroxidase complex method. Brain sections were washed in 0.1% bovine serum albumen (Jackson ImmunoResearch Laboratories, West Grove, PA; cat# 001-000-162) in PBS (PBS+BSA) for 3 × 10 min at room temperature (r.t.). Sections were then incubated in 0.3% Triton X-100 in PBS+BSA containing the primary antibody, a monoclonal anti-acetylcholinesterase (anti-AChE [HR2]; AbCam, Cambridge, MA, USA; cat# ab2803; diluted 1:5000) for 24 h at 4°C. Sections were then washed in PBS+BSA for 3 × 10 min at r.t. before being incubated in PBS+BSA containing the secondary antibody, a biotinylated goat anti-mouse IgG (Vector laboratories, Burlingame, CA; cat# BA-9200; diluted 1:200) for 45 min. at r.t. Sections were processed using the ABC Elite kit (Vector laboratories, Burlingame, CA; cat# PK-6100; diluted 1:50), washed again in PBS+BSA for 3 × 10 min at r.t., and finally reacted with a solution of 3% H_2_O_2_, 73μg/ml 3,3'-diamino benzidine tetrahydrochloride in 0.05 M Tris, pH 7.6. Sections were then mounted on charged glass slides and allowed to dry overnight. Slides were dehydrated through 100% ethanol, cleared in Histoclear II (Electron Microscopy Sciences, Hatfield, PA; cat# 64111), and coverslipped using DPX Mountant (Sigma-Aldrich, St. Louis, MO; 06522). Images were captured under 10x magnification and automatically stitched together using Adobe Photoshop (CS5; San Jose, CA).

## Results

### Probabilistic Tractography Across Subjects

**Fig 3:**
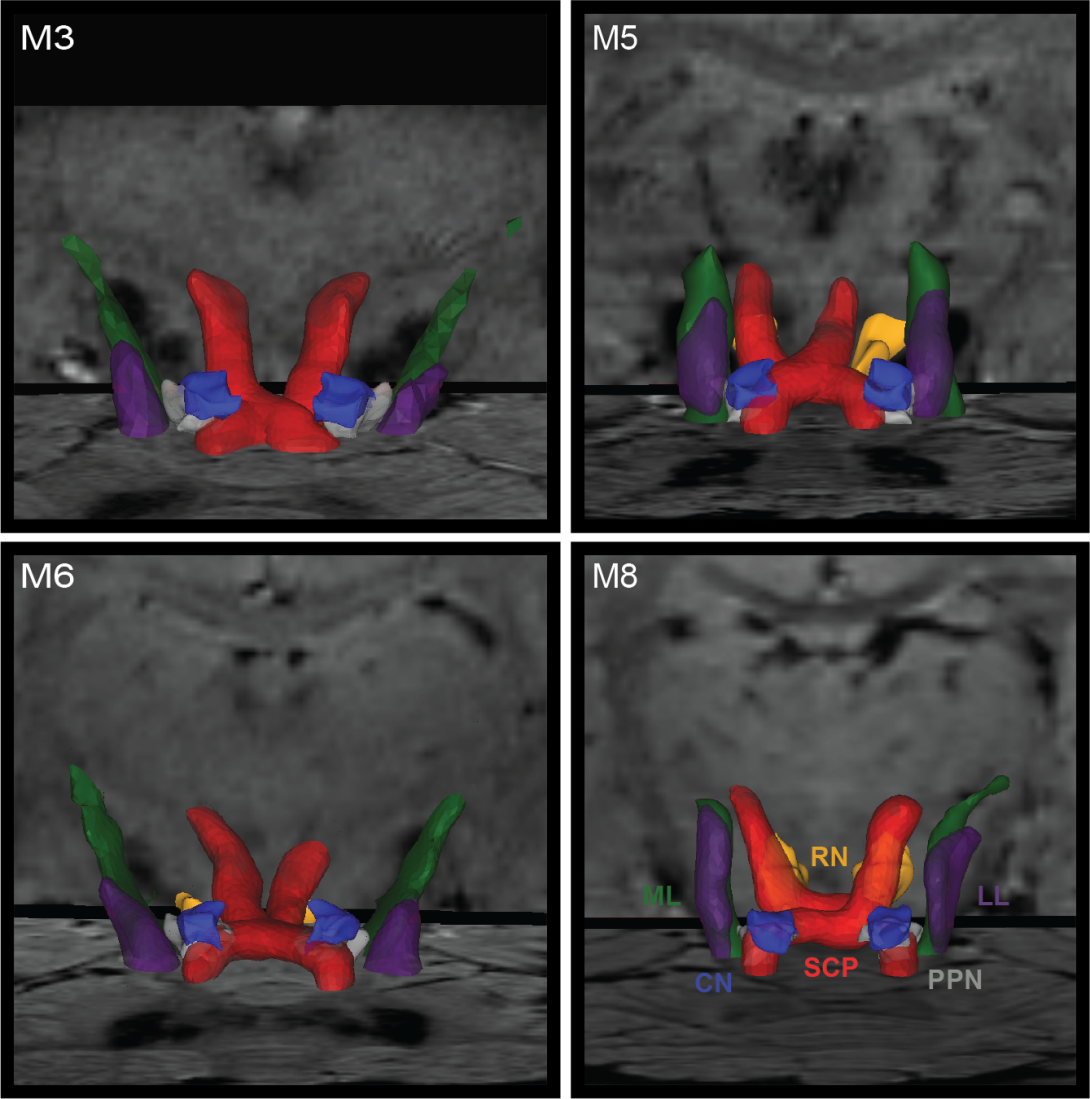
Brainstem tractography displayed with warped nuclei in four rhesus macaques. Tractography is shown for SCP (red), LL (purple), and ML (green). SCP is shown to course through PPN (grey) and around RN (gold).

Tractography in the brainstem can be difficult to calculate accurately in cases of small feature sizes and high density of divergent fiber tracts that can include decussations. In four subjects (8 hemispheres), structural SWI scans and warped slices were used as a guide to define seed points and waypoints for probabilistic tractography analysis of the SCP, ML, and LL (Fig 3). Whereas a single region of interest for seed and way points was sufficient to identify ML and LL fiber tracts, additional waypoints were needed to delineate SCP at its decussation. Additionally, in 4 of 8 hemispheres, inclusion of an RN waypoint was necessary to obtain a fiber tract that targeted the cerebellar-receiving area of thalamus. While fiber tract consistency was evident across hemispheres, subject-specific variability was also present (Fig 3). The fiber tractography results were compared to atlas-warped reconstructions of fiber tracts transformed into diffusion tractography space. The reconstruction overlap within the brainstem between tractography and nonlinear atlas warping, was 45±4% (mean ± std.dev.) for SCP, 45±24% for ML, and 39±13% for LL, which reflected consistent albeit slight misalignments in which tract borders identified through the nonlinear warping approach were rendered slightly caudal to the diffusion tractography volume reconstructions.

### Visualization in Brainstem with 7T Imaging

**Fig 4:**
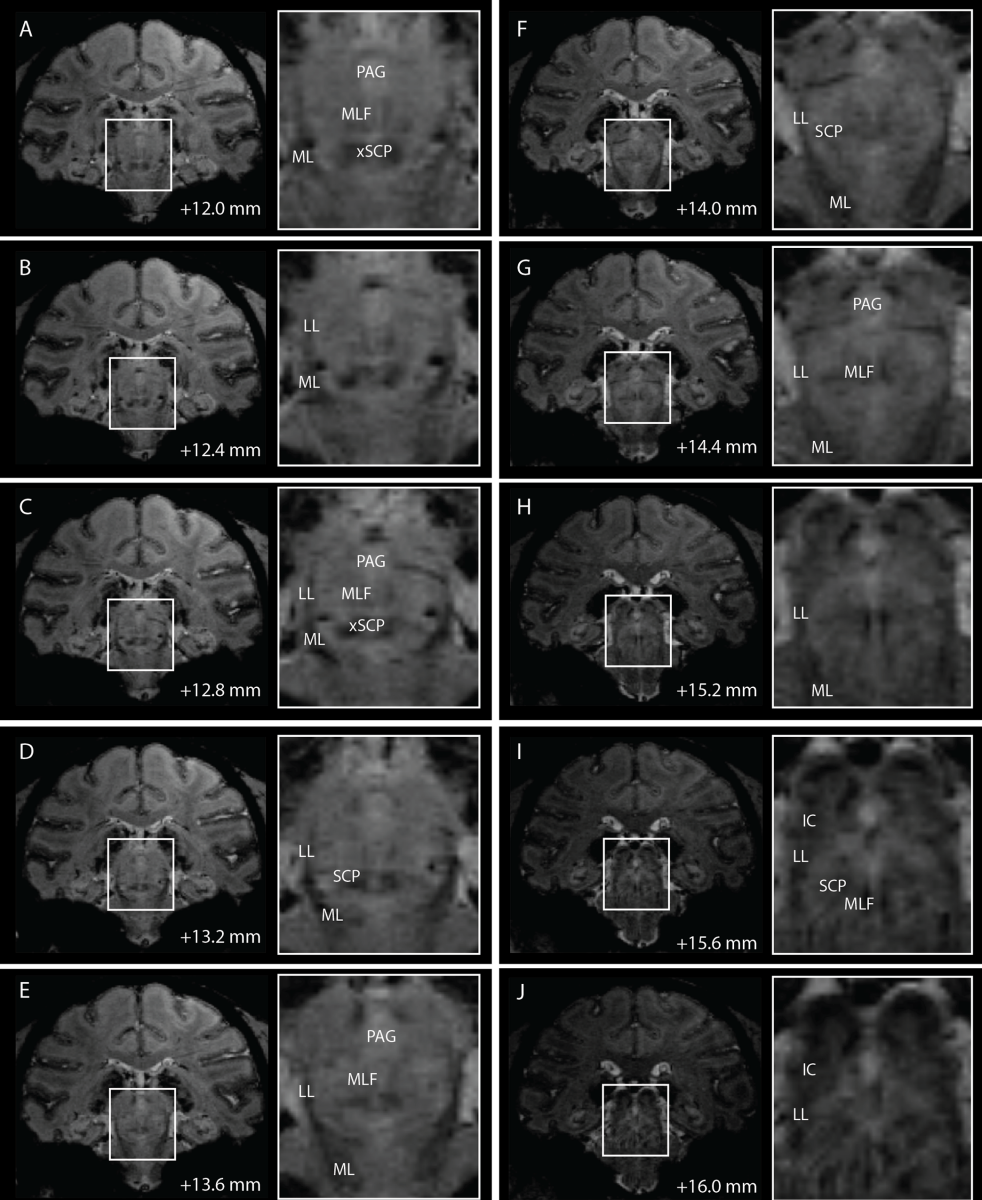
Coronal slices of 7T SWI of the brainstem in subject M2. Distance of each slice from the midline crossing of the anterior commissure are noted at the bottom of each coronal slice.

While nuclei such as the red nucleus were visible on T2-W images, little contrast was present within other brainstem regions with either T1-W or T2-W imaging. Conversely, high field 7T SWI using the sequences described above provided improved contrast to visualize borders of several brainstem nuclei and white matter tracts that are relevant to neurosurgical targeting procedures (Fig 4).

**Fig 5:**
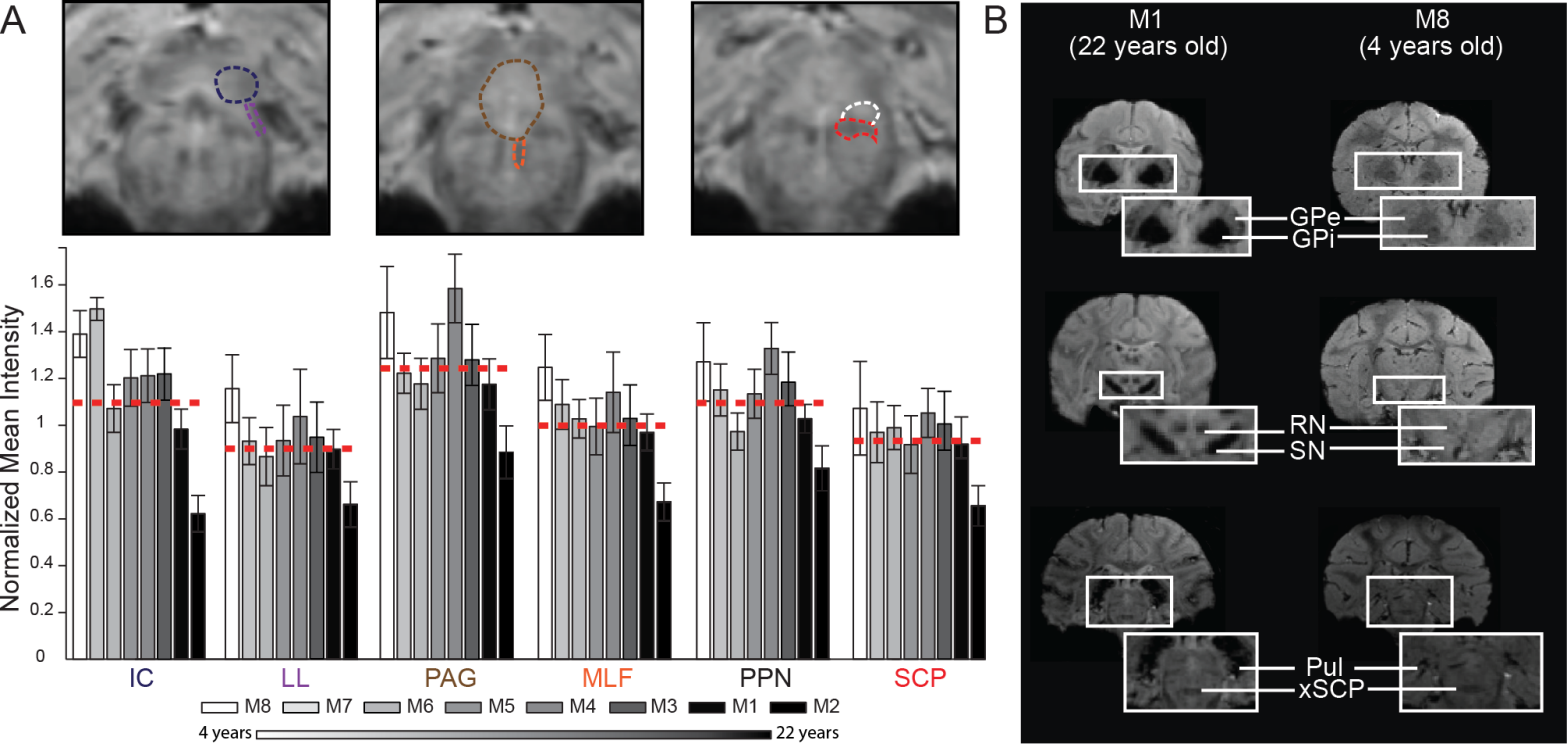
Comparisons of SWI normalized pixel intensity across brainstem regions and rhesus macaques. (A) Analysis of three paired brainstem nuclei and adjacent fiber tracts, each corresponding to an investigational target for DBS therapy. Pixel intensity values were calculated by averaging pixel intensities for each region (0 = black, 255 = white) from the raw SWI scans and dividing by the average pixel intensity values of the anterior commissure about the midline. Columnar intensity values are plotted in age order with white being the youngest and black being the oldest subject. (B) Example of age-dependent SWI pixel intensity across basal ganglia, thalamus, and brainstem structures.

Regional variation in brainstem pixel intensity was quantified for three DBS target nuclei (IC, PAG, and PPN) and compared to the pixel intensity within a white matter tract adjacent to each target (LL, medial longitudinal fasciculus (MLF), and SCP, respectively) (Fig 5A). Anatomical borders for each nucleus and fiber tract were defined from the warped atlas reconstructions in each primate. Normalized mean pixel intensity was then calculated by dividing the average pixel intensity for each region by the average pixel intensity of the anterior commissure about the midline for each subject. The anterior commissure was chosen for normalization since its intensity was not found to correlate with age, based on a linear regression analysis (r^2^ = 0.0735, slope = 0.6057, p = 0.4839). In almost all cases, white matter tracts displayed a lower mean intensity ratio than nuclei adjacent to them within the brainstem, where a lower ratio represents a more hypointense region on the susceptibility-weighted image.

Additionally, SWI data showed age-dependent normalized mean intensities for nuclei and white matter tracts, with older animals exhibiting greater hypointense imaging within the brainstem (Fig 5A). For example, M8 (4 years) had a ratio of 1.07 for SCP, while M2 (22 years) had a ratio of 0.656. Overall, correlation analysis (Spearman’s ρ, df = 6, N = 8, p<0.05) showed that normalized mean intensity for two out of the three fiber tracts had a statistically significant dependence on age (MLF: r = -0.8571, p = 0.0065, and SCP: r = -0.7143,p = 0.0465) as did the inferior colliculus (IC: r = -0.8095, p = 0.0149) but not LL (r = -0.6190, p = 0.1017), PPN (r = -0.5238, p = 0.1827) and PAG (r = -0.5238, p = 0.1827). These age-dependent intensity findings were found to extend to other subcortical nuclei as well, including the RN, globus pallidus (internal and external segments), and substantia nigra (Fig 5B).

### Histological Confirmation of 7T Imaging in Brainstem

**Fig 6:**
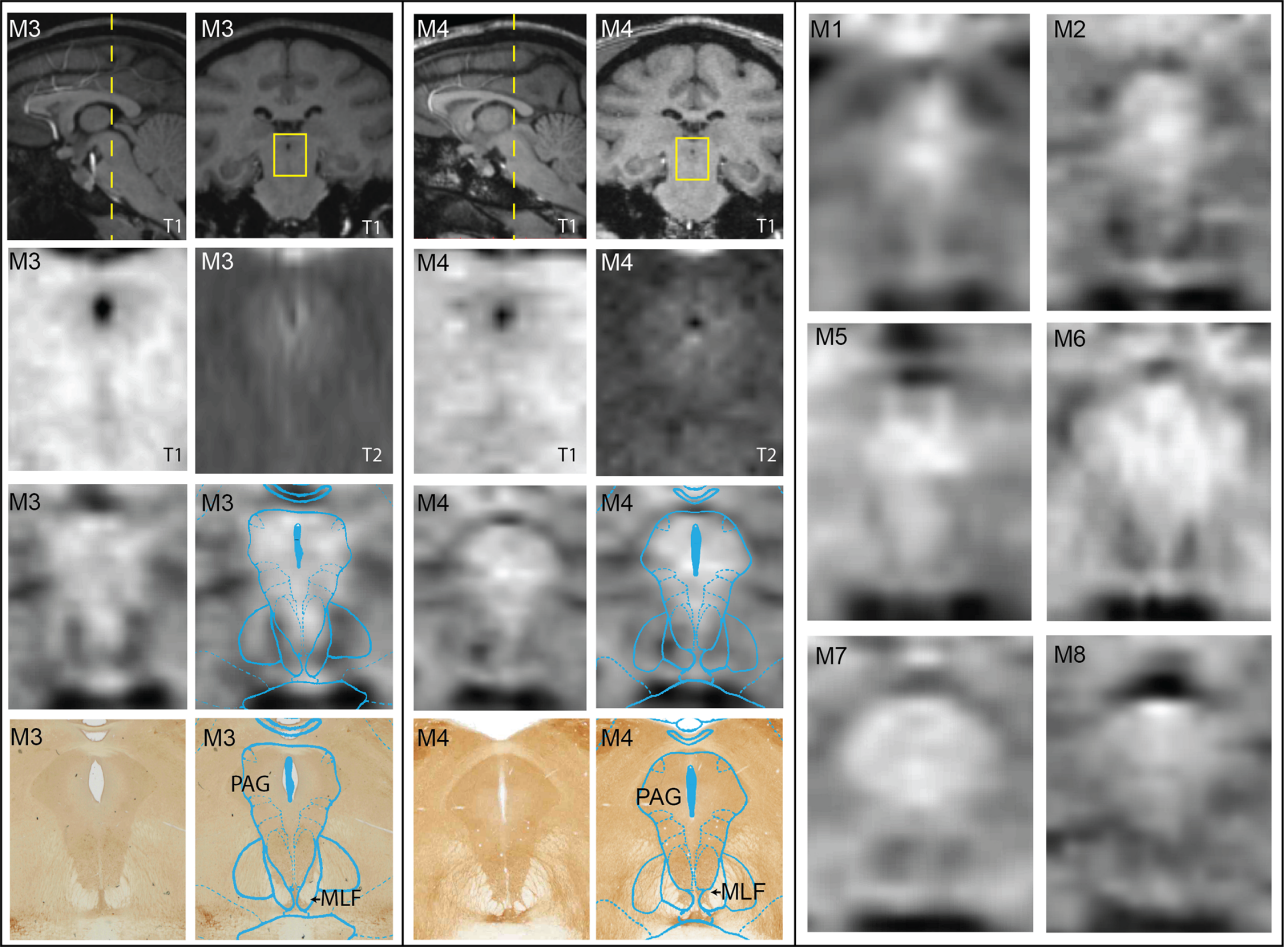
Imaging PAG with comparisons between MRI modalities and immunolabeled histology. Coronal SWI, T1, and T2 images were matched to corresponding histological slices stained with AChE from the same animal (M3 and M4). The corresponding warped atlas was overlaid on both the SWI and the histology. On the right, matched coronal SWI slices are shown for all other animals. Histograms for all coronal MRI slices were not altered, but stretched to encompass the entire spectrum (0–255).

To confirm the location of nuclei and fiber tracts resultant from the atlas-based warping algorithm and tractography, post-mortem histology was performed on M3 and M4. In the case of PAG, little contrast was visible in either the T1-W or T2-W MRI, whereas SWI scans showed consistent hyperintensity of the PAG in comparison to adjacent fiber tracts including the MLF and deep white layer of the superior colliculus in all animals (Fig 6). While all primates displayed subject-specific variability, the hyperintensity of PAG was consistent with the AChE-labeled histological sections and the warped atlas results of the PAG in both M3 and M4.

**Fig 7:**
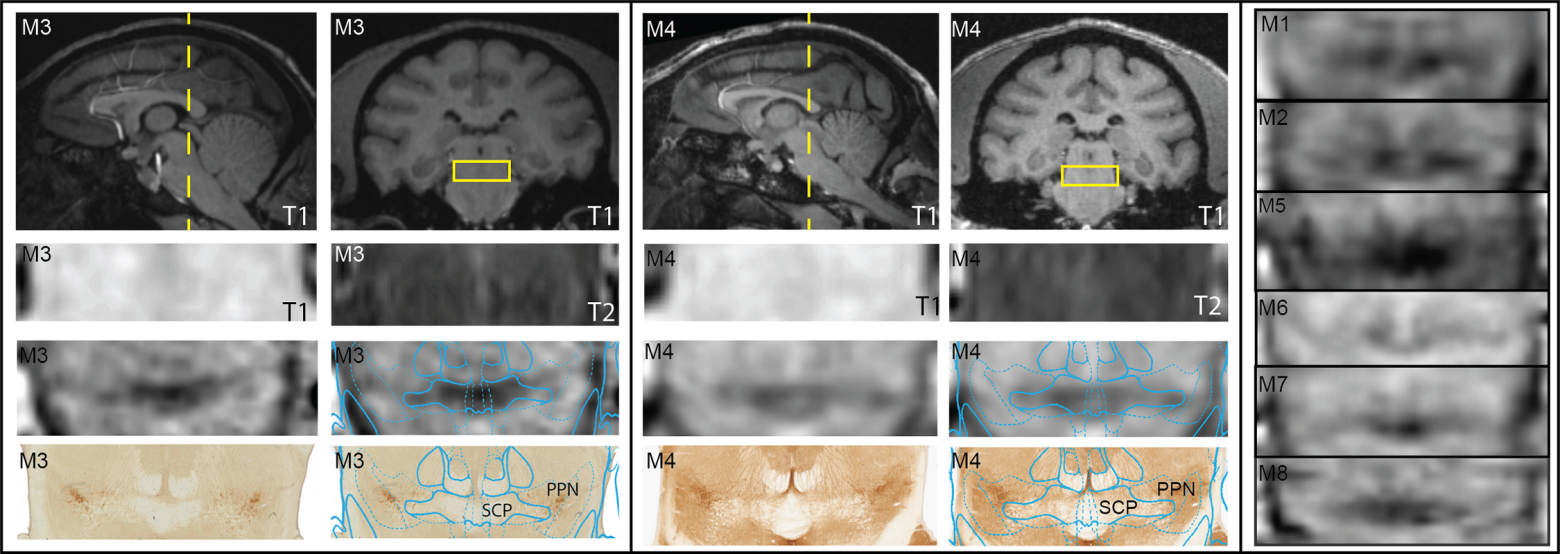
Imaging PPN with comparison between MRI modalities and immunolabeled histology. Coronal SWI, T1, and T2 MRI were matched to corresponding histological slices stained with AChE from M3 and M4. The corresponding warped atlas was overlaid on both the SWI and the histology. Matched coronal SWI slices are shown for all other animals for comparison. Histograms for all coronal MRI slices were not altered, but stretched to encompass the entire spectrum (0–255).

Fig 7 shows 7T imaging results of the PPN region and its adjacent fiber tracts. Similar to the PAG region, there was no meaningful contrast in either the T1-W or T2-W MRI at this level of the brainstem. AChE labeling identified cholinergic cells within PPN in the histological sections and further demarcated adjacent fiber tracts as regions with no labeling. Cholinergic cell labeling was especially notable within and lateral to SCP, which was consistent with a gradation from a hypointense core of the SCP to a diffuse hyperintense region on the lateral border of SCP. Relative hypointense distributions were also found to be consistent with the LL and ML fiber tracts.

**Fig 8:**
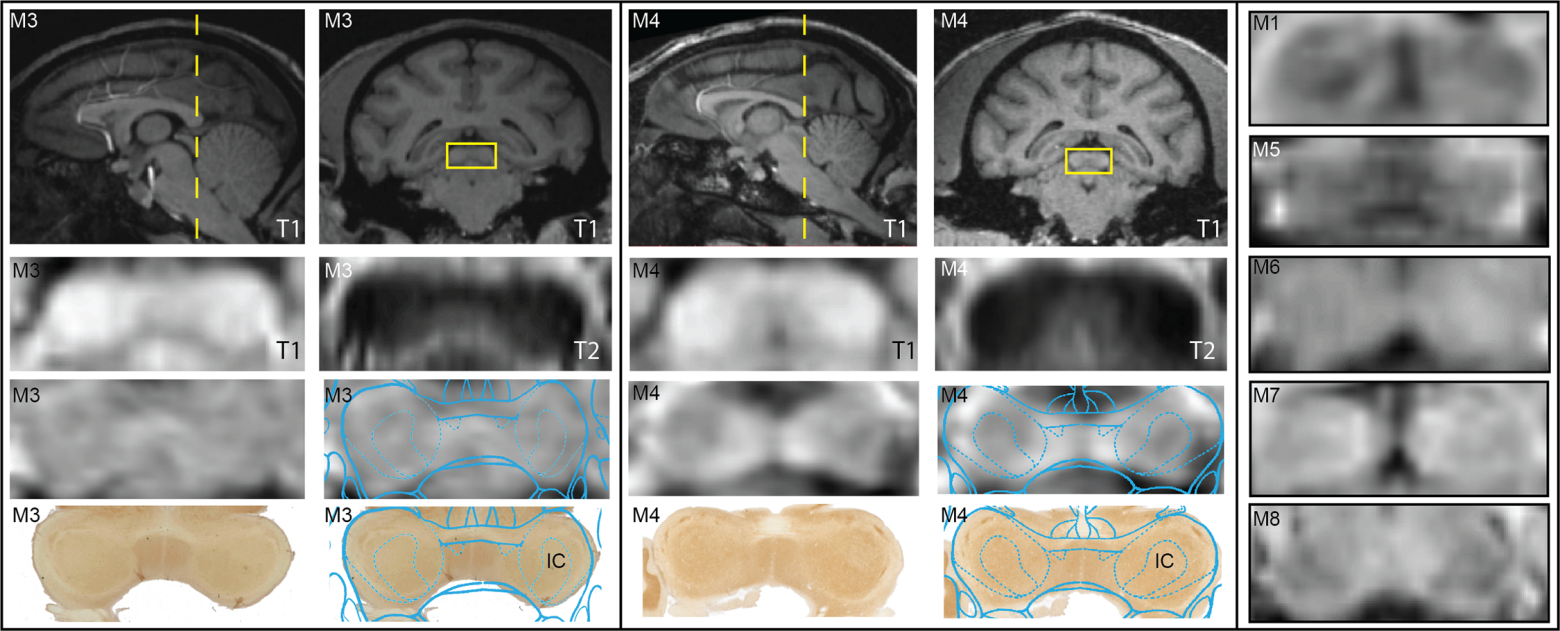
Imaging IC with comparison between MRI modalities and immunolabeled histology. Coronal SWI, T1, and T2 MRI were matched to corresponding histological slices stained with AChE from M3 and M4. The corresponding warped atlas was overlaid on both the SWI and the histology. Matched coronal SWI slices are shown for all animals for comparison (except M2 in which the 7T MRI scans did not extend to the level of the IC). Histograms for all coronal MRI slices were not altered, but stretched to encompass the entire spectrum (0–255).

The warped atlas and histology results were also compared with coronal SWI of the IC (Fig 8). T1-W and T2-W images, while able to demarcate IC, had no variation in contrast within the structure. SWI in eight rhesus macaques (posterior SWI in M2 was not imaged) showed consistency across subjects in visualizing the external borders of IC as well as a fairly robust consistency for most subjects in demarcating the central nucleus of IC as a region of relative hypointensity.

## Discussion

Here, we developed a multimodal imaging approach using 7T MRI to identify nuclei and fiber tracts *in vivo* within the nonhuman primate brainstem and verified the interpretation of these imaging results with post-mortem histology. This approach to subject-specific imaging, which consisted of SWI at 7T coupled with a nonlinear brain atlas warping algorithm and high angular diffusion weighted imaging with probabilistic tractography, has potential for greatly improving imaging of the brainstem for neurosurgical targeting applications. A similar approach, used by Lenglet et al. [32], combined high-field 7T SWI and DWI in humans to visualize white matter pathways within and between the basal ganglia and thalamus. In this case, manual segmentation coupled with probabilistic diffusion tractography in FSL allowed for delineation of the nigrostriatal, subthalamopallidal, pallidothalamic, and thalamostriatal pathways in humans. Our approach expands upon these techniques with the addition of a nonlinear brain atlas warping algorithm, the application of probabilistic tractography to the brainstem region, and importantly histological confirmation of the imaging results.

DBS within the brainstem is currently under investigation for treatment of parkinsonian freezing of gait (PPN) [5, 6, 10–12], relieving central pain (PAG) [7], and restoring hearing (IC) [8]. While these regions of the brainstem are certainly difficult to target given their depth and high degree of vascularization [61], the lack of contrast within the brainstem with standard MRI sequences can further limit subject-specific targeting of DBS leads. Visualization of both nuclei and surrounding fiber tracts is important for DBS targeting, as fiber tracts play an important role in accurately interpreting therapeutic outcomes for targets in the basal ganglia [62–65] as well as targets in the brainstem [9]. In PPN DBS, for example, adjacent fiber pathways can be activated including SCP, ML, and LL resulting in potential motor coordination problems, paresthesias, and auditory disturbances, respectively [60, 66–69]. Similarly, stimulation in the region of the dorsal PAG for relief of pain can lead to adverse sensory side effects, nausea, contralateral piloerection, and cold sensations in the face [70]. For auditory midbrain stimulation targeted to the central nucleus of the IC, targeting errors can result in poor activation of the underlying tonotopy and potential induction of side effects including paresthesia, dizziness, facial twitch, and temperature sensation [71].

Imaging in nonhuman primates, as opposed to humans, provided a means to both further the translational potential of animal models of DBS and histologically corroborate the interpretation of the high-field imaging data that would otherwise be difficult to accomplish in humans. It is important to note, however, that the methods developed in this study to better visualize nuclei and white matter tracts in the brainstem are directly transferable to human MRI and DTI, as was demonstrated by Lenglet, et al. when visualizing the connectivity patterns of the human basal ganglia [32]. Animal models have been used as investigational tools for many studies including deep brain stimulation in the brainstem [8, 72–74] as they are useful for dissecting mechanisms of therapy and optimizing means for delivering therapy. As part of these studies, accurate targeting of DBS leads is critical towards generating data with meaningful and translational outcomes. Using non-human primates also enabled histological analysis to relate labeling within fixed tissue with 7T SWI contrast within the brainstem. These results indicated that for SWI in the brainstem, white matter tracts had a relative hypointensity compared to gray matter regions and further that the degree of image intensity depended upon age with older animals exhibiting greater relative hypointensity in the brainstem. Interestingly, the observed image hypointensity in brainstem fiber tracts may not necessarily apply to other brain regions, such as the globus pallidus, red nucleus, and substantia nigra, which exhibited strong hypointensity relative to their surrounding white matter tracts.

While many regions within the brainstem had visible contrast differences to adjacent brainstem structures, other regions were more difficult to demarcate. Here we employed a nonlinear warping algorithm to tailor a brain atlas to each subject’s imaging data to identify these structures. Current nonlinear warping methods utilize 3D warping based on the cortex, ventricles, or 3D points [75–77] or incorporate both MRI and other imaging modalities such as PET [78, 79] or CT [80]. Deformations, which rely on matching cortical surfaces and relegating this information to the deformation of deep structures, may not provide adequate deformation because it is not clear that cortical morphology is relevant to morphology of deep structures [81]. The richness of seed point information in the 2D slices, especially at 7T, could provide more accurate results [82]. This process also enabled cropping the cortex from the images and focusing on the brainstem to perform local deformations. The warping algorithm identified nuclei consistent with histological results, which allowed for the accurate placement of seed points for running the tractography analysis. Probabilistic diffusion tractography using high angular diffusion weighted imaging [32] also has strong value especially when fiber tracts run in close proximity to one another and decussate as was the case for the superior cerebellar peduncle. Care must be taken when interpreting these results, as probabilistic diffusion tractography does not visualize the actual tracts but determines the most likely direction of the fiber tracts based on a measure of diffusion along many directions. However, the combination of the tractography and the precise anatomical borders of the tracts obtained from the SWI could provide a means to more accurately define fiber tracts and their directions within the brain, which would be especially useful for computational models of DBS [9, 52, 63, 83, 84].

Certain limitations should be considered upon interpretation of the results in this study. First, it should be noted that the data set used seven females and one young male rhesus macaque. The choice of these subjects was based on being able to place the receiver coils closer to the brain than would otherwise be possible in older male rhesus macaques with large cranial musculature. Additionally, the warped nuclei and fiber tracts are limited to the regions delineated in the atlas, and these demarcations among nuclei and fiber tracts in the atlas are discrete, whereas some anatomical boundaries are not well defined, as shown for the interdigitation of PPN and SCP. Furthermore, there is a discrepancy between the voxel size of the SWI (0.33–0.4 mm) and the DTI (1 mm). These factors provide some context for the slight variations between the atlas-based fiber tract identification methods and diffusion tractography results. Inherent variability of tracts between animals further necessitates the use of subject-specific techniques that are not based on atlases. Although Duchin, et al. showed that the use of 7T compared to 3T has negligible differences in distortion in the region of the midbrain, further studies are needed to examine the issue of geometric distortion in the brainstem at high fields and develop methods for their corrections. Other considerations include the use of a brain atlas generated from a single rhesus macaque [85], the method used to loft 3D objects from the warped slices, and the probabilistic nature of the fiber tractography calculation. Additionally, 3D rendering of histology-based fiber tracts [86] may be used to validate tractography and warping methods. While high-field *in vivo* MRI is poised to help demarcate regions within the brainstem pre-surgically for neurosurgical targeting procedures, it cannot account for other factors that contribute to targeting inaccuracies during surgery including probe deflection, brain shift, and microdrive imprecision [87].

Together, these multi-modal imaging techniques (7T SWI, 7T DWI and probabilistic tractography, and nonlinear brain atlas warping) provide subject-specific methods to more precisely identify regions of the brainstem and provide an enabling set of tools to assist in the neurosurgical procedures targeting the brainstem.

## Supporting Information

S1 ARRIVE Guidelines Checklist The Animal Research: Reporting in vivo experiments Guidelines Checklist. (DOCX)
