# Supplementary material for: In Vivo 7T MRI of the Non-Human Primate Brainstem
Source: PLoS One. 2015 May 12;10(5):e0127049. doi: 10.1371/journal.pone.0127049 (PMC4428864; doi:10.1371/journal.pone.0127049)

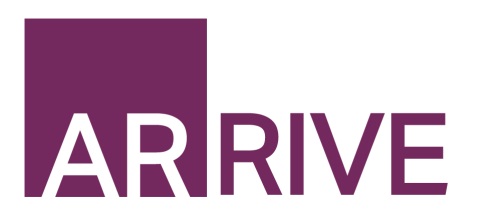


The ARRIVE Guidelines Checklist

Animal Research: Reporting In Vivo Experiments

Carol Kilkenny^1^, William J Browne^2^, Innes C Cuthill^3^, Michael Emerson^4^ and Douglas G Altman^5^

*^1^The National Centre for the Replacement, Refinement and Reduction of Animals in Research, London, UK, ^2^School of Veterinary Science, University of Bristol, Bristol, UK, ^3^School of Biological Sciences, University of Bristol, Bristol, UK, ^4^National Heart and Lung Institute, Imperial College London, UK, ^5^Centre for Statistics in Medicine, University of Oxford, Oxford, UK.*

|  | | ITEM | RECOMMENDATION | Section/ Paragraph |
| --- | --- | --- | --- | --- |
| 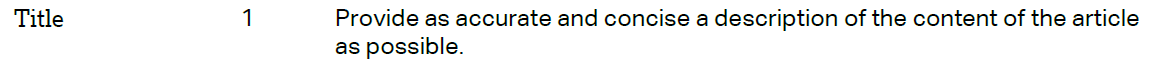 | | | Title |  |
| 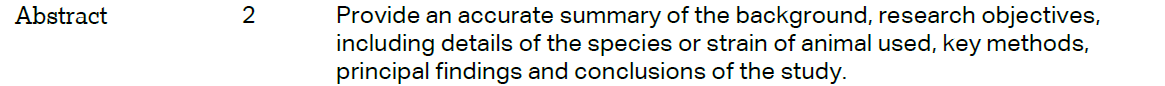 | | | Abstract |  |
| INTRODUCTION | | |  |  |
| 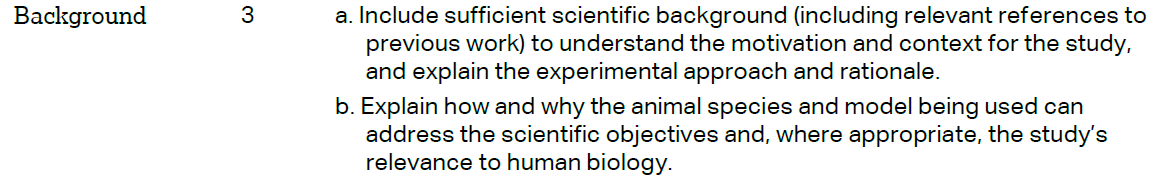 | | | Paragraphs1-4 |  |
| 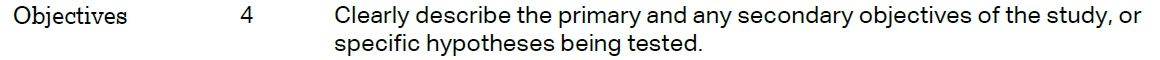 | | | Paragraph 4 |  |
| METHODS | | |  |  |
| 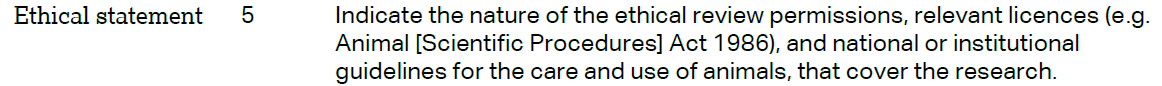 | | | Paragraph 1 |  |
| 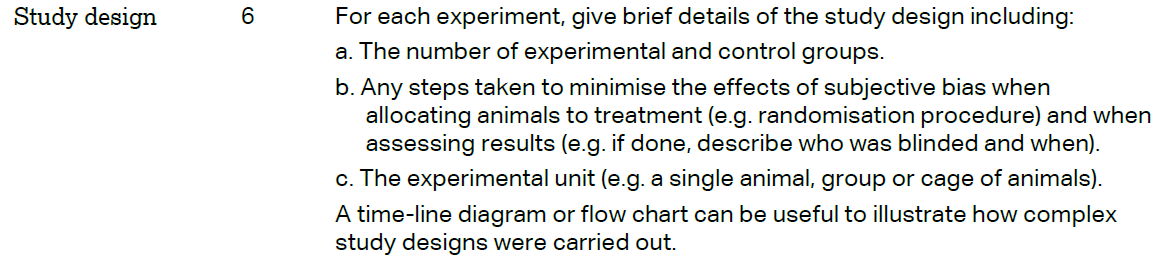 | | | Paragraphs 1 and 2 |  |
| 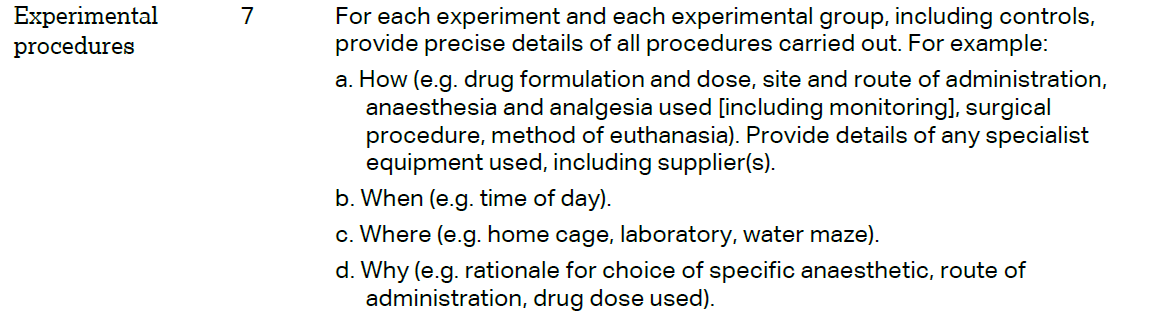 | | | Paragraphs 1, 2, and 7 |  |
| 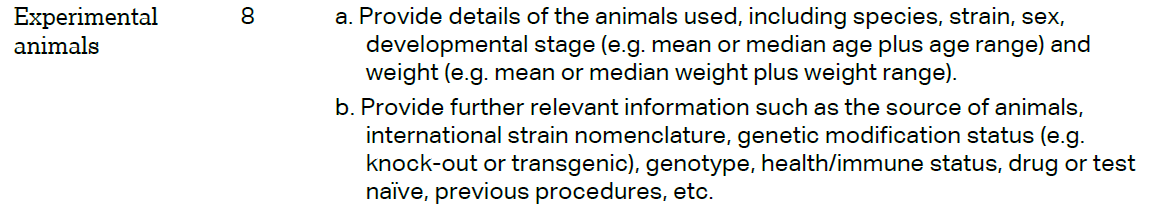 | | | Paragraph 1 and Table 1 |  |

The ARRIVE guidelines. Originally published in *PLoS Biology*, June 2010^1^

| 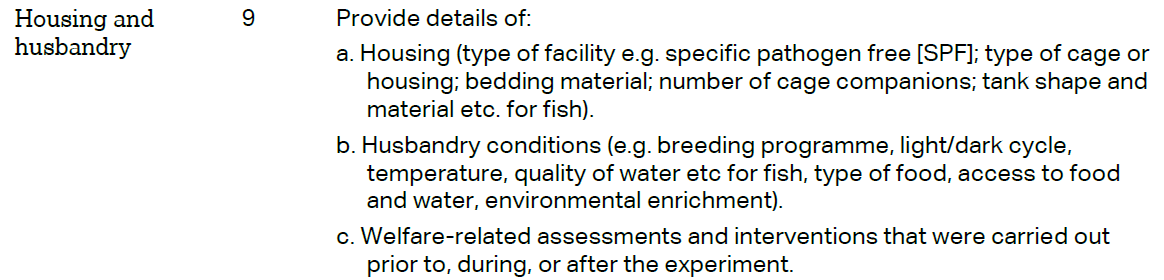 | Paragraph 1 | |
| --- | --- | --- |
| 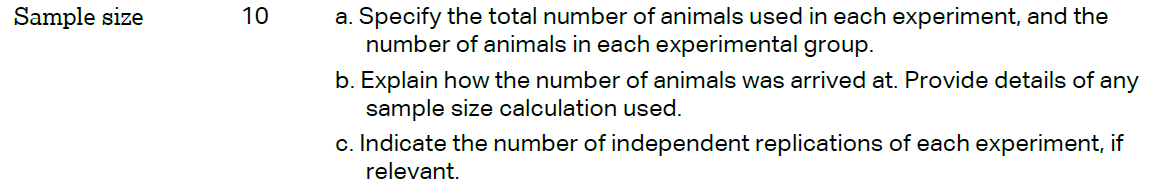 | Paragraph 1 and Table 1 | |
| 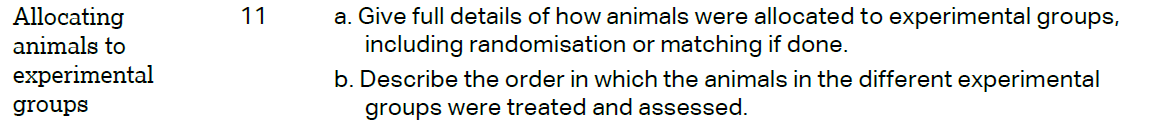 | Paragraph 1 | |
| 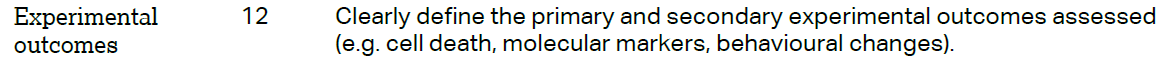 | Paragraphs 2-8 | |
| 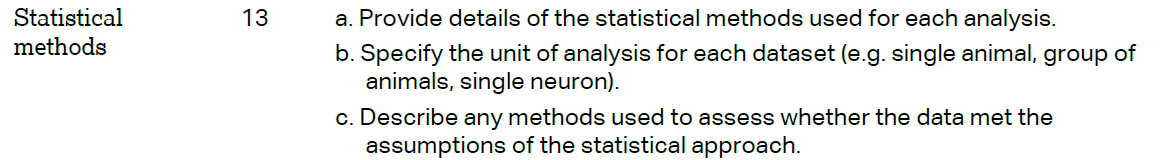 | Methods: Paragraphs 3-6, Results: Paragraphs 1-4. | |
| RESULTS |  | |
| 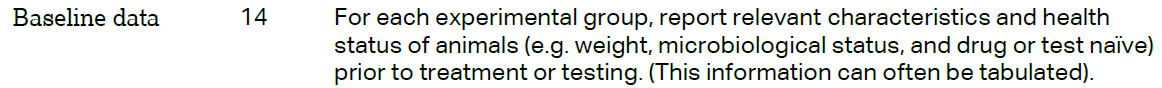 | Methods: Paragraph 1 | |
| 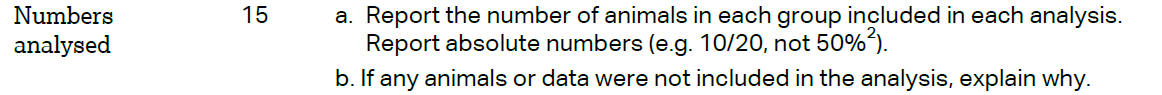 | Methods: Paragraphs 1/7, Results: Paragraph 1 | |
| 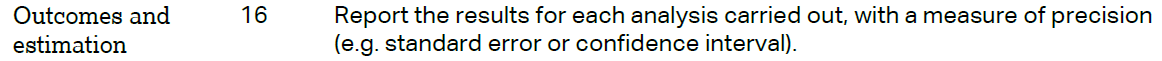 | Paragraphs 1-7 | |
| 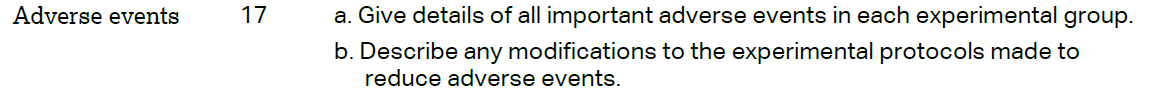 | Methods: Paragraph 1 | |
| DISCUSSION |  | |
| 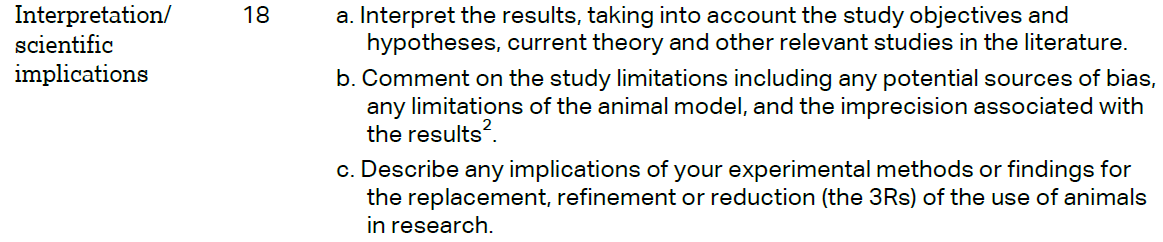 | Paragraphs 1-6 | |
| 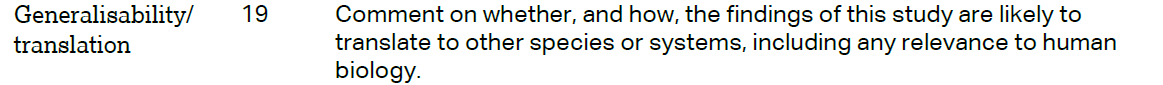 | Paragraphs 1, 3, 5, and 6 | |
| 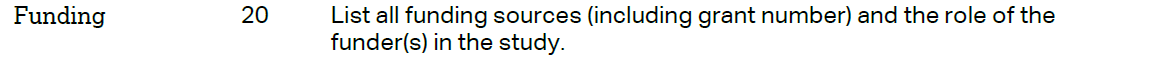 | | Financial Disclosure |


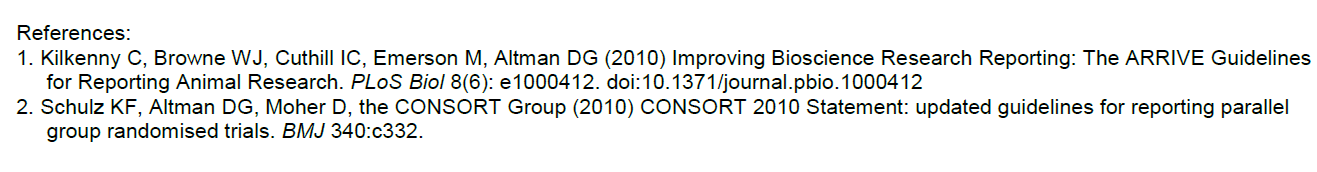

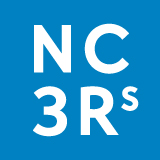

Supplement: S1 ARRIVE Guidelines Checklist — (DOCX) [file pone.0127049.s001.docx]
